# Mesenchymal Stem Cells Exhibit Regulated Exocytosis in Response to Chemerin and IGF

**DOI:** 10.1371/journal.pone.0141331

**Published:** 2015-10-29

**Authors:** J. Dinesh Kumar, Chris Holmberg, Silvia Balabanova, Lyudmyla Borysova, Ted Burdyga, Robert Beynon, Graham J. Dockray, Andrea Varro

**Affiliations:** 1 Department of Cell and Molecular Physiology, Institute of Translational Medicine, University of Liverpool, Crown St, Liverpool, United Kingdom; 2 Department of Biochemistry, Institute of Integrative Biology, University of Liverpool, Crown St, Liverpool, United Kingdom; Georgia Regents University, UNITED STATES

## Abstract

Mesenchymal stem cells (MSCs) play important roles in tissue repair and cancer progression. Our recent work suggests that some mesenchymal cells, notably myofibroblasts exhibit regulated exocytosis resembling that seen in neuroendocrine cells. We now report that MSCs also exhibit regulated exocytosis. Both a G-protein coupled receptor agonist, chemerin, and a receptor tyrosine kinase stimulant, IGF-II, evoked rapid increases in secretion of a marker protein, TGFβig-h3. The calcium ionophore, ionomycin, also rapidly increased secretion of TGFβig-h3 while inhibitors of translation (cycloheximide) or secretory protein transport (brefeldin A) had no effect, indicating secretion from preformed secretory vesicles. Inhibitors of the chemerin and IGF receptors specifically reduced the secretory response. Confocal microscopy of MSCs loaded with Fluo-4 revealed chemerin and IGF-II triggered intracellular Ca^2+^ oscillations requiring extracellular calcium. Immunocytochemistry showed co-localisation of TGFβig-h3 and MMP-2 to secretory vesicles, and transmission electron-microscopy showed dense-core secretory vesicles in proximity to the Golgi apparatus. Proteomic studies on the MSC secretome identified 64 proteins including TGFβig-h3 and MMP-2 that exhibited increased secretion in response to IGF-II treatment for 30min and western blot of selected proteins confirmed these data. Gene ontology analysis of proteins exhibiting regulated secretion indicated functions primarily associated with cell adhesion and in bioassays chemerin increased adhesion of MSCs and adhesion, proliferation and migration of myofibroblasts. Thus, MSCs exhibit regulated exocytosis that is compatible with an early role in tissue remodelling.

## Introduction

The recruitment by peripheral tissues of bone marrow derived mesenchymal stem cells (MSCs) is a crucial component in tissue responses to damage, inflammation and progression to cancer [1,2,3]. The tissue microenvironment in turn provides the conditions required for differentiation of MSCs into different cell types. A variety of chemokines is thought to be involved in the process of MSC recruitment and there is also a requirement for matrix metalloproteinases (MMPs) in facilitating their transendothelial migration from the circulation into peripheral tissues [4,5,6]. In addition to the putative roles of MSCs in tissue regeneration there are potential therapeutic applications of MSCs in immune- and inflammatory modulation and as delivery vectors [7,8,9].

All cells possess the capacity for secretion, and the secretomes of MSCs have attracted increasing attention [10,11,12,13]. Protein secretion in neurons, endocrine and exocrine cells can occur via either the constitutive pathway or the regulated pathway where exocytosis of storage (usually dense cored) vesicles occurs in response to secretagogues following an increase in intracellular calcium [14,15]. In other cell types including mesenchymal cells, protein secretion is often considered to be constitutive, although it is recognised that there is capacity for regulated secretion in these cells [16].

Insulin-like growth factors are produced by gut myofibroblasts and stimulate proliferation and migration of both these cells and epithelial cells [17]. In addition, they may stimulate protein secretion from the regulated pathway in mesenchymal cells including myofibroblasts [18]. Chemerin (tazarotene induced gene 2, TIG2; retinoic acid receptor responder 2, RARRES2) is an 18kDa chemokine-like protein that acts at a G-protein coupled receptor, ChemR23 (chemokine-like receptor 1, CMKLR1) [19,20], and is capable of stimulating secretion of MMP-2 by MSCs [4]. In view of the relatively rapid secretion of MMP-2 by MSCs in response to chemerin we hypothesized that MSCs exhibit regulated exocytosis. We now report that IGF and chemerin stimulate calcium-dependent release of a range of proteins from preformed secretory vesicles in MSCs, that they stimulate increased intracellular calcium albeit with different time-courses, and that increased secretion leads to an altered microenvironment capable of changing adhesion of MSC themselves and adhesion and migration of other cell types.

## Materials and Methods

### Cells

Human bone marrow derived mesenchymal stem cells were used at passages 3–12 in their undifferentiated state as previously described [4]; cells were CD105, CD166, CD29, CD44, α-SMA and vimentin positive and were CD14, CD34, CD45, cytokeratin and desmin negative; up to passage 12 these cells exhibited adipocyte, osteocyte and chondrocyte differentiation in adipocyte, osteocyte and chondrocyte differentiation media (Lonza, Cambridge, UK) [4]. Primary human normal oesophageal myofibroblasts had been generated from transplant donors as previously described [4,18].

### Cell Culture

MSCs were maintained in an undifferentiated state in MSCGM (Lonza) containing basal medium and MSC growth supplements. Cells were maintained at 37°C in 5% v/v CO_2_. Myofibroblasts were cultured as previously described [21]

### Secretion assays

Cells (10^6^) were plated in 10cm dishes, incubated overnight, then washed 3 times with 10ml sterile PBS, and incubated in 5ml serum-free media (SFM) for 1 h followed, unless otherwise stated, by stimulation for 30 min with 100ng.ml^-1^ chemerin (R&D Systems Inc., Oxfordshire, UK), 100ng.ml^-1^ recombinant human GF-II, 50ng.ml^-1^ IGF-I (R&D Systems Inc.) or 1μM ionomycin (Sigma-Aldrich, Poole, UK). In some experiments cells were preincubated for 30 min with 10μg.ml^-1^ brefeldin A (Epicentre Biotechnologies, Cambio Ltd, Cambridge, UK), 10μg.ml^-1^ cycloheximide (Sigma-Aldrich), 3.2μM AG1024 (Calbiochem) or 1μM CCX832 (ChemoCentryx, Mountain View, CA). After stimulation, medium was centrifuged (800g 4°C, 7 min) and stored at -80°C.

### Protein extraction

Proteins in media were concentrated using StrataClean resin (Agilent Technologies Ltd., Wokingham, UK)[22] and cellular proteins extracted using RIPA lysis buffer containing 1% v/v Phosphatase Inhibitor Cocktail set II, and 1% v/v Protease Inhibitor Cocktail Set III, EDTA-Free (Calbiochem). Total protein was determined using the DC Protein Assay kit (Bio-Rad Lab Inc., Hemel Hempstead, UK).

### Conditioned media

MSCs (1.5 x 10^6^ cells) were plated in T-75 falcon flasks and maintained at 37°C in 5% v/v CO_2_ for 24 h in full media (FM). Cultures were then washed 3 times with sterile PBS, followed by 1 h in serum free (SF) medium which was than replaced with 15ml fresh SF media for 30 min with or without or chemerin (100ngml^-1^). Conditioned media (CM) were collected, centrifuged (7min, 800 x g, 4°C) and aliquots were stored at -80°C until further use.

### Intracellular Ca^2+^

Cells in glass bottom dishes were loaded with Fluo-4 acetoxymethyl ester (Invitrogen, UK, 15μM, dissolved in DMSO with 0.1% pluronic F-127) for 15min at 24°C and then transferred to indicator-free solution for ≥30min. The dishes with Fluo-4 loaded cells were transferred to the stage of an inverted Olympus microscope. Superfusion of cells was performed by applying a positive pressure, valve-controlled, flow via a 100μm diameter tip attached to a 3-d mechanical manipulator (Narishige, Japan) which allowed positioning of the superfusion tip in the desired region of the dish. The solution was removed by suction at the other end of culture dish. All experiments were performed at 30°C. We used a Nipkow disc, confocal microscope (Ultraview, Perkin Elmer, Waltham, MA), connected to an iXon cooled charge-coupled device camera (Andor Technology, UK). Andor Technology iQ or iQ2 data acquisition software was used for 2- and 3-dimensional confocal imaging of Fluo-4 loaded MSCs. Images were collected at 33 frames per second using dry (×10, NA 0.42) or, water immersion (x40, NA 1.2) objectives (Olympus).

### SILAC labelling and protein identification

Cells were cultured in media supplemented with either ^12^C_6_ lysine and ^12^C_6_ arginine (light label), or ^13^C_6_ lysine and ^13^C_6_ arginine (heavy label) for at least 6 population doublings. Cells were then plated (10^6^ cells per 10cm diameter dish) and grown for 24 h in full media, washed three times with PBS, and cultured in serum-free medium for 1 h followed by 5 ml fresh serum free medium containing IGF-II (100ng.ml^-1^). Medium was collected after 30 min, centrifuged (800 x g for 7 min), concentrated by adding StrataClean resin (10μg.ml^-1^), mixing for 1 min and then separating by centrifugation. The resin was washed three times with 25mM ammonium bicarbonate, resuspended in 25mM ammonium bicarbonate and denatured by addition of 0.05% (w/v) RapiGest (Waters) and incubated at 80°C for 10 min. Samples were reduced by 3mM DTT at 60°C for 10 min, and alkylated by 9mM iodoacetamide at room temperature for 30 min. They were then digested by addition of sequencing-grade trypsin in a roughly 50:1 protein:trypsin ratio and incubated at 37°C for 18 h. Digested peptides were collected in a fresh tube by centrifugation at 14,000 x g for 10 min, followed by a further elution with 50μl 0.5M NaCl. Samples were then desalted using C18 ZipTips (Millipore), dried, and resuspended in 20μl 3% acetonitrile, 0.1% formic acid. Samples were processed in triplicate on a Nano-Acquity (Waters) reverse phase HPLC system in-line with an LTQ Orbitrap Velos (Thermo). SILAC data were searched and analysed using MaxQuant 1.1.1.36 against the human IPI database v3.68. The following software settings were used; Orbitrap instrument setting, doublet SILAC experiment with a maximum of 2 labelled amino acids per peptide; variable modifications were methionine oxidation and N-terminal acetylation, fixed modification was carbamidomethyl cysteine; the trypsin/P enzyme was selected with a maximum of 1 missed cleavage; MS/MS tolerance was 0.5 Da; the number of top MS/MS peaks per 100 Da was set to six. FDR was set to 0.01. The data were searched against the human IPI database v3.68, as well as a reversed database and a contaminant database downloaded together with the MaxQuant software. Protein data were then further analysed using t-tests in Perseus (MaxQuant) to identify those proteins with a ratio significantly different from unity. Proteins were reported based on the assignment in minimum of two of the triplicates with at least two tryptic peptides with a confidence >99% and a local FDR calculated using the PSPEP algorithm of <1%. Proteins exhibiting an increased abundance of 1.2 in the presence of IGF-II were considered to exhibit stimulated secretion and those exhibiting no difference or less than 1.2- fold change in response to IGF were defined as “constitutive” in keeping with previous studies [18]. A list of UniProt Accession numbers of proteins in the stimulated MSC secretome was uploaded in *Protein Analysis Through Evolutionary Relationships* (PANTHER) classification system, ver9.0 and compared with a reference *H*. *sapiens* dataset. Binomial statistical tests [23] extracted significantly enriched protein classes, biological process, molecular functions and pathways as described [24].

### Western blotting

Media or cell extracts prepared in RIPA buffer containing protease and phosphatase inhibitors were resolved by SDS-PAGE electrophoresis and processed for western blotting as previously described [17] using antibodies to MMP-2, TGFβigh-3, macrophage migration inhibition factor (MIF), decorin, insulin growth factor binding protein (IGFBP)-7 (R&D Systems Inc.), lumican and secreted protein acidic and rich in cysteine (SPARC) (Santa Cruz Biotechnology, Inc., Heidelberg, Germany). Typically media samples equivalent to the secretory product of 2 x10^5^ cells and cell extracts equivalent to 6–8 x10^4^ cells were loaded per lane.

### Immunocytochemistry

Cells were paraformaldehyde(PFA)-fixed (4% w/v), permeabilised with 0.2% Triton X-100 in PBS (PBS-T) for 30 min and processed for immunocytochemistry as previously described [25] using primary antibodies to MMP-2 (902MP; R&D Systems Inc.), TGFβig-h3 (AF-2935; Cell Signaling, Massachusetts, USA), MIF (AF-289; R&D Systems Inc.) and SPARC (SC 25574; Santa Cruz Biotechnology, Inc.) followed by incubation with the appropriate fluorescein or Texas Red labelled secondary antibodies raised in donkey (711-075-152 and 705-585-147; Jackson Immunoresearch, Soham, UK), and mounted with Vectashield^®^ containing DAPI (Vector Laboratories, Peterborough, UK). Slides were viewed using a Zeiss Axioplan-2 microscope (Zeiss Vision, Welwyn Garden City, UK). Images were captured using a JVC-3 charge-coupled device camera at 40X magnification

### Transmission electron microscopy (TEM)

Cells (80% confluency in 10 cm dishes) were processed for TEM using a resin pellet embedding protocol as published previously [26]. Multiple images were taken of 5–10 cells from four MSCs. The analysis of images for the presence of dense core secretory vesicles was conducted by an observer who was blind to the identity of the cells. Size of the secretory vesicles was measured using analySIS Pro 3.2 (Build 678) program.

### Bioassays

MSCs (1x10^5^) were plated in full media in 12-well plates and incubated with or without chemerin (100ng.ml^-1^) for 30 min at 37°C. Primary human oesophageal myofibroblasts (1x10^5^) were plated in 12-well plates and incubated with chemerin-conditioned MSC media alone, and with CCX832 (1μM, generous gift from ChemoCentryx, MountainView, Ca, USA) for 30 min at 37C. Fixing, staining of adhered cells and absorbance was done as previously described [27]. Migration of myofibroblasts was studied in 8 μm pore BD cell culture control inserts (BD Bioscience, Oxford, UK) as described previously [21] employing chemerin-conditioned MSC media with or without CCX832. In addition, proliferation of myofibroblasts was measured by 5-ethynyl-2´-deoxyuridine (EdU) incorporation, according the manufacturer’s instructions, using Click-iT^®^ EdU Alexa Fluor^®^ 488 Imaging Kit (Life Technologies Ltd., Paisely, UK) [4]. Counting of EdU stained cells were done at 10x magnification using a Zeiss Axioplan-2 microscope.

### Statistics

Unless otherwise stated results are expressed as mean ± standard error of the mean; Student’s t-test or analysis of variance (ANOVA; Systat Software Inc., Hounslow, London, UK) were used as appropriate to determine statistical significance of results and considered significant at P < 0.05.

## Results

### Human MSCs exhibit regulated TGFβig-h3 secretion

**Fig 1:**
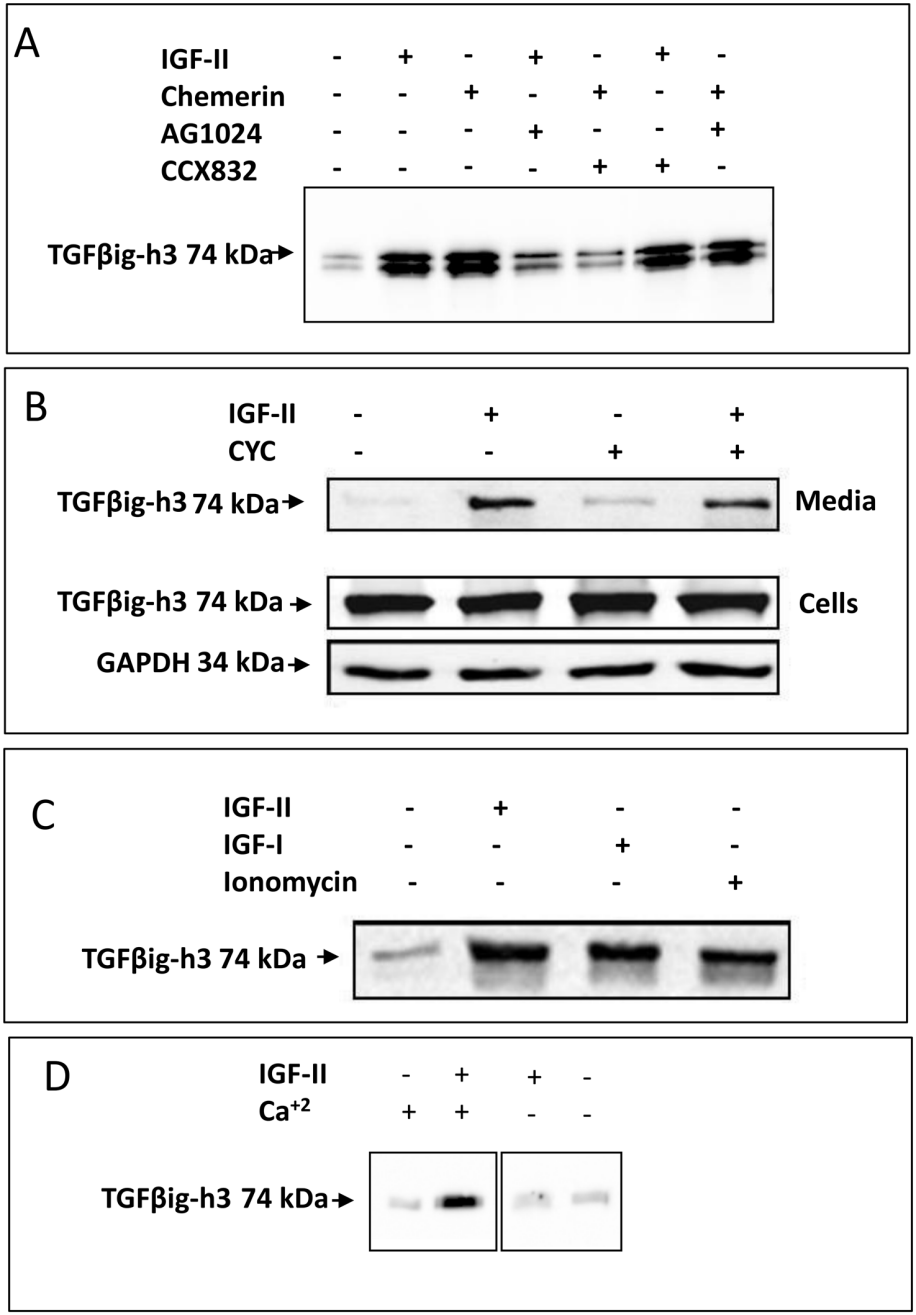
Secretion of TGFβig-h3 is stimulated by chemerin and IGF. *A*. Western blot analysis of TGFβig-h3 in media from MSC cells shows stimulation by chemerin and IGF-II and inhibition by CCX832 and AG1042, respectively. *B*. Stimulated secretion of TGFβig-h3 is maintained after cycloheximide treatment. TGFβig-h3 abundance in cell extracts was unchanged (middle panel); GAPDH was used as a loading control for the cell extracts (bottom panel). *C*. The calcium ionophore, ionomycin (1μM) stimulated TGFβig-h3 secretion comparable to IGF-I (50ng.ml^-1^) and IGF-II (100ng.ml^-1^). *D*. In calcium-free medium stimulated secretion in response to IGF-II is inhibited.

Since TGFβig-h3 secretion has previously been shown to be a robust marker of regulated exocytosis in myofibroblasts [18] we first characterised TGFβig-h3 secretion in MSCs. Cells exhibited increased secretion of TGFβig-h3 in response to stimulation with either IGF-II or chemerin typically in bands of 74 and 68 kDa that varied between MSC samples [18] (Fig 1A). Responses to chemerin were inhibited by the ChemR23 receptor antagonist CCX832 but not by an IGF receptor tyrosine kinase inhibitor AG1024; conversely, the response to IGF-II was inhibited by AG1024 but not CCX832 (Fig 1A). The response was evident in several different MSC lines (S1 Fig). IGF-II stimulated secretion of TGFβig-h3 was still present after preincubation with cycloheximide compatible with secretion from pre-existing cellular stores (Fig 1B). Similarly, in the presence of brefeldin A, which inhibits constitutive secretion by blocking ER to Golgi transport, the secretory responses were preserved pointing to release from a pre-existing store of secretory vesicles (S1 Fig). The calcium ionophore, ionomycin, also stimulated TGFβig-h3 secretion from MSCs that was comparable to that IGF-I and IGF-II indicative of a response via Ca^2+^ dependent exocytosis (Fig 1C), while the response to IGF-II was attenuated in the absence of extracellular calcium (Fig 1D).

### Calcium responses to chemerin and IGF

**Fig 2:**
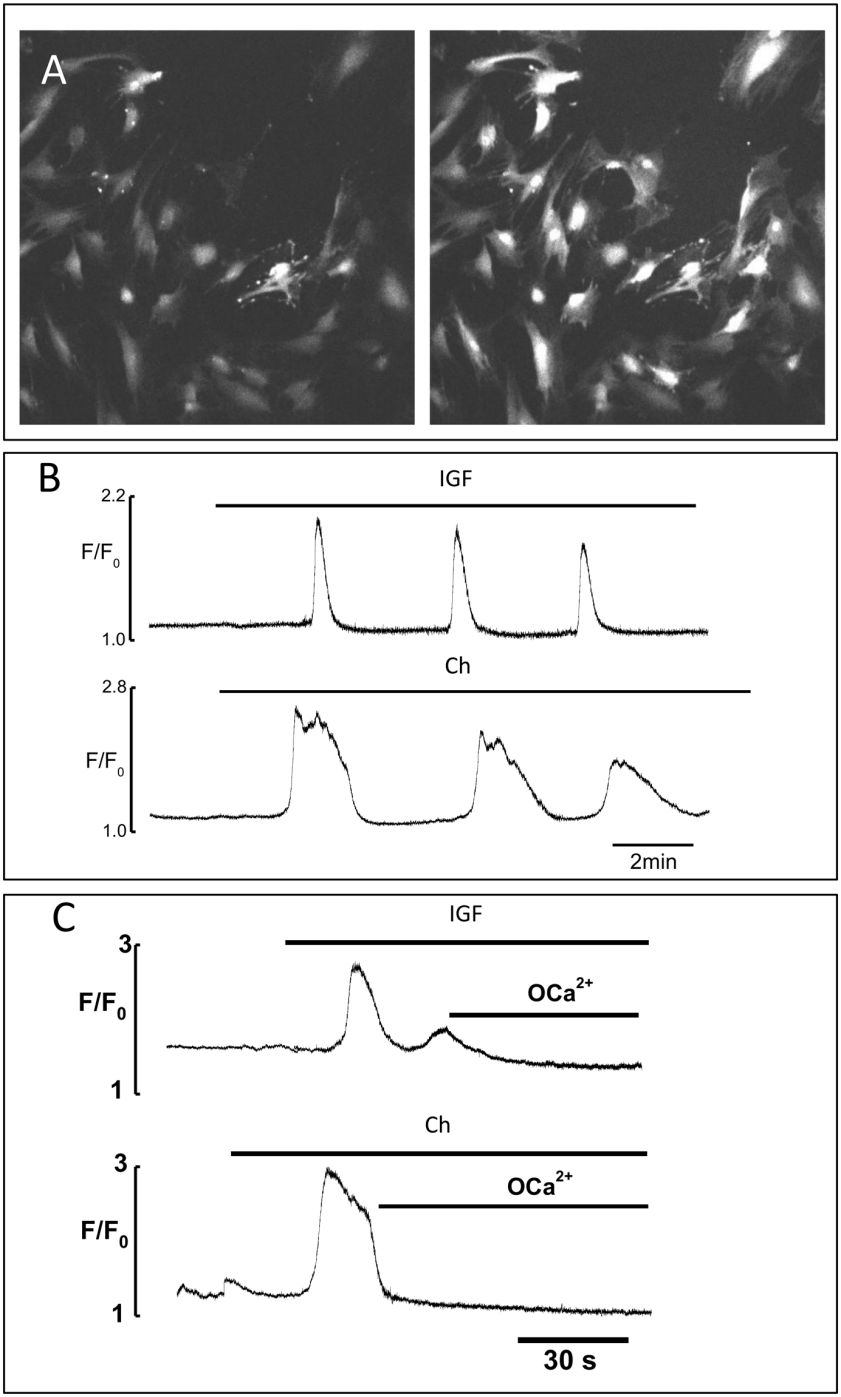
Effects of IGF and chemerin on Ca^2+^ signalling in MSCs. *A*. Images of Fluo-4 loaded MSCs taken in the absence (left) and the presence (right) of chemerin (100nM), respectively. *B*. IGF-II (100 ng.ml^-1^, top) and chemerin (100nM, Ch) induce Ca^2+^ oscillations in Fluo-4 loaded cells. *C*. Relaxation of Ca^2+^ transients induced by chemerin or IGF-II after removal of external Ca^2+^ (Ca^2+^ free solution with 2mM EGTA).

The data suggest that there is calcium dependent regulated secretion from MSCs. To determine whether IGF-II and chemerin increase intracellular Ca^2+^ in these cells, we first established that they could be loaded with Fluo-4 and that on loading they retained their morphology (S2 Fig). Application to the media of both IGF-II and chemerin initiated intracellular Ca^2+^ oscillations (Fig 2). IGF-II-initiated Ca^2+^ oscillations were observed in 20–30% of cells, and chemerin-evoked oscillations were seen in 70–90% of cells (Fig 2B). The duration of Ca^2+^ oscillations induced by chemerin was more than 3 times longer than that induced by IGF-II (Fig 2B). In both cases, Ca^2+^ oscillations were instantly and fully abolished by removal of external Ca^2+^ (Fig 2C). The data suggest that both agents increase Ca^2+^ permeability and induce Ca^2+^ oscillations consistent with a role in regulating exocytosis.

### Identification of proteins in the secretomes of MSCs

In order to define the range of extracellular proteins that were secreted by MSCs in response to acute stimulation, we examined by SILAC the MSC secretome following stimulation with IGF-II for 30min (S3 Fig; S1 Table). A total of 64 proteins, quantified on the basis of at least two tryptic peptides in at least two replicates, were considered to be “secretory” when searched using the Universal Protein Resource (UniProt) database. The majority of these were soluble secretory proteins (n = 59). Interestingly, nearly all (62) were identified as secreted by the regulated secretory pathway since their abundance was at least 20% higher in IGFII-treated compared with untreated samples.

**Fig 3:**
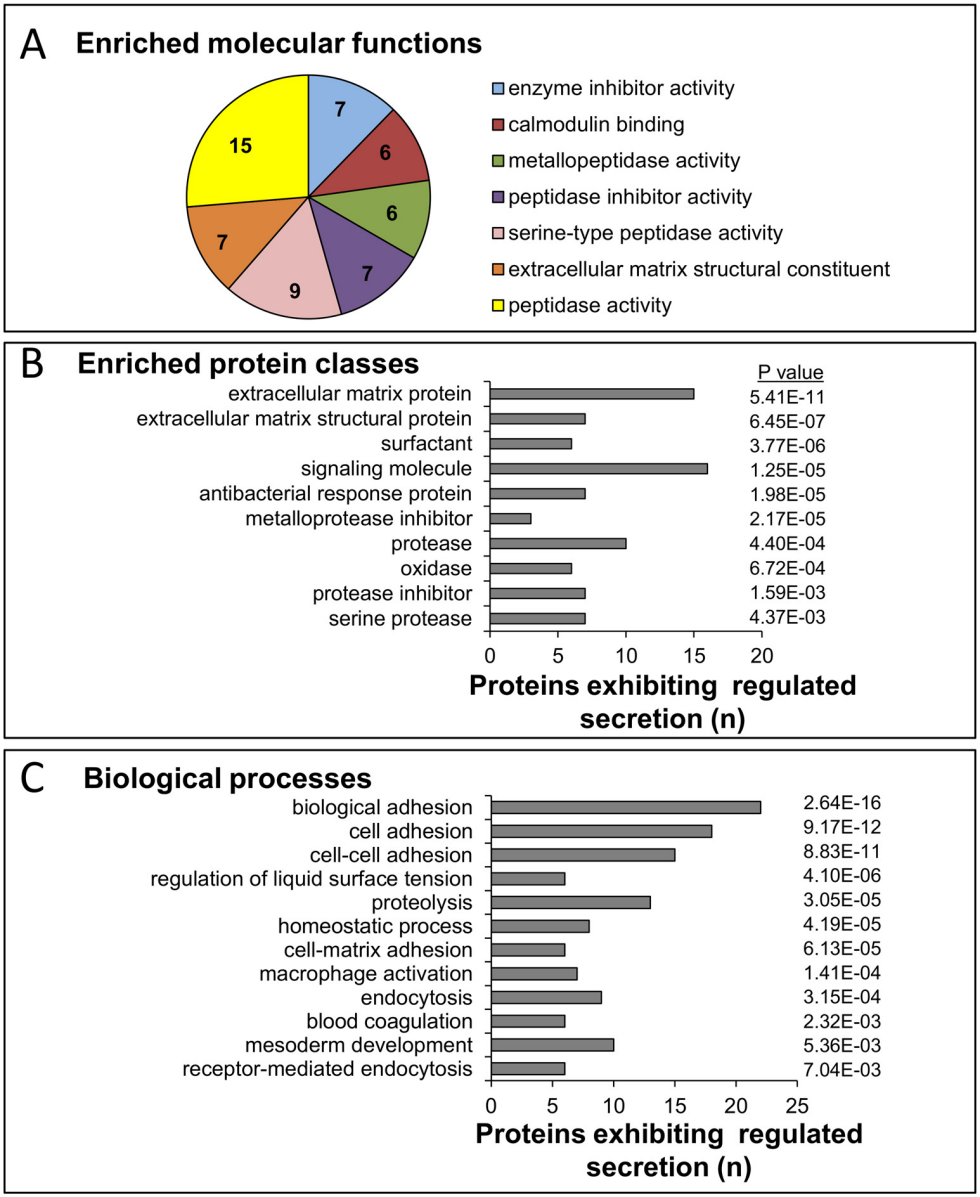
Enriched molecular functions, protein classes and biological processes in the MSC secretome. *A*, The main molecular functions shown by PANTHER for 62 proteins in the MSC secretome identified as “secreted” and as exhibiting increased abundance after IGF-II treatment. *B*. Enriched protein classes, *C*. Enriched biological processes. P, probability.

Gene ontology, functional enrichment and pathway analysis of the 64 proteins were performed using PANTHER with a significance threshold cut-off of p<0.05 for proteins identified as constituting the IGF-II-regulated MSC secretome. Analysis of enriched molecular functions showed 7 significantly enriched groups, the top being *peptidase activity*, represented for example by tissue inhibitor of metalloproteinases (TIMP) -1, -2, -3, and MMP-2 (Fig 3A). A total of 12 protein classes were significantly over-represented of which the top three were extracellular matrix proteins, signalling molecules and proteases (Fig 3B). Gene ontology analysis showed 17 significantly over-represented biological processes with cell adhesion, cell matrix adhesion and cell-cell adhesion as the top three (the top 12 are listed in Fig 3C). The only significant over-represented (>5) pathway was the integrin signalling pathway which was represented by 11 proteins on the regulated secretome list (p = 2.29E-08).

### Validation of SILAC identifications in the mesenchymal stromal cell secretome

**Fig 4:**
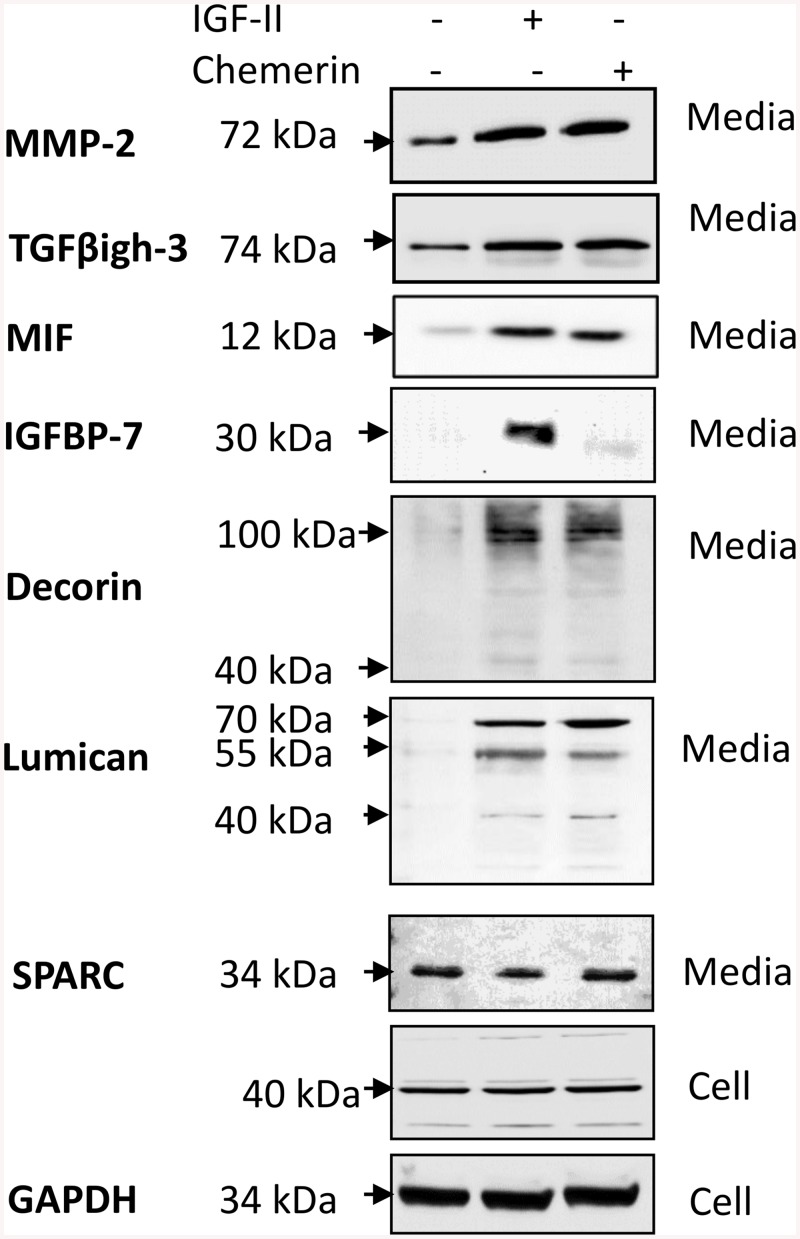
Western blot validation of proteomic studies of the MSC secretome. MSCs were treated with chemerin or IGF-II and media or cell extracts probed by western blot for proteins identified by SILAC. Six proteins that exhibited increased secreted in proteomic studies were also increased in western blots response to IGF (MMP-2, TGFβig-h3, MIF, IGFBP-7, decorin and lumican) and all except one (IGFBP-7) were also stimulated by chemerin. SPARC was not identified as exhibiting stimulated exocytosis in proteomic studies and neither did it respond to IGF-II or chemerin in western blot studies. Cellular content of SPARC and GAPDH was not influenced by IGF-II or chemerin.

In order to validate the proteomic identification of the regulated MSC secretome, we performed western blot on a subset of secreted proteins selected on the basis of representation of different functional classes and including an example of a putative constitutively secreted protein (SPARC). All of 6 proteins (TGFβig-h3, MMP-2, MIF, decorin, lumican, IGFBP-7) exhibiting an increased relative abundance after IGF-II treatment in SILAC studies were also increased in western analysis (Fig 4), although curiously, one (IGFBP-7) exhibited a response to IGF-II but not chemerin. In contrast, SPARC was not stimulated in either the SILAC analysis or in western blots (Fig 4). The secretion of selected proteins exhibiting IGF-II or chemerin-stimulated exocytosis was rapid with detectable responses after just 5 min of stimulation (S4 Fig), was resistant to BFA treatment (S5 Fig) but was sensitive to AG1024 or CCX832, respectively (S6 Fig).

### Immunocytochemistry

**Fig 5:**
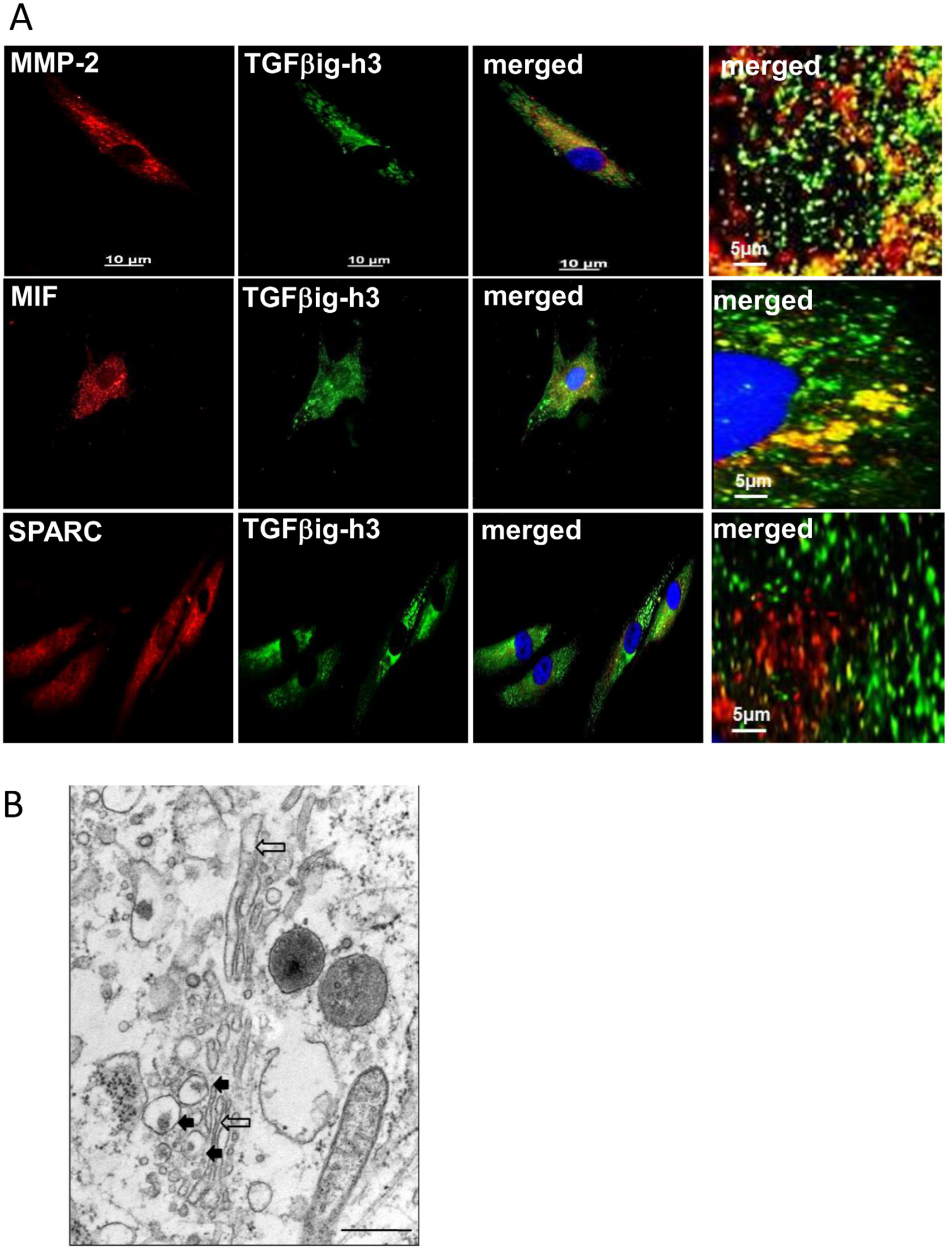
Localisation of proteins in secretory vesicles. *A*. localisation of TGFβig-h3 (green) and MMP2, MIF or SPACR (red) in MSCs revealed by immunocytochemistry. In each case there are images for the red channel, green channel, a marge of these two images, and finally a merged image at higher magnification. There are clear examples of TGFβig-h3 and either MIF or MMP-2 in similar vesicular populations (yellow or orange), while TGFβig-h3 and SPARC are localised to distinct vesicular structures (red or green vesicular structures). Nuclei stained blue with DAPI. Scale bars 5 or 10 μm. *B*. TEM identifies dense core secretory vesicles in MSCs. representative photomicrograph taken at a magnification of 60,000x show dense core secretory vesicles (closed arrows) in MSCs the Golgi apparatus is clearly visible (open arrows). Scale bar 500nm.

We reasoned that proteins exhibiting regulated exocytosis *eg* TGFβig-h3, MIF and MMP-2, would be co-localised in the same secretory vesicles while SPARC would be localised to a separate vesicle population. Immunocytochemistry revealed overlapping localisations of TGFβig-h3 with both MMP-2 and MIF seen in Fig 5A as orange or yellow punctate staining. In comparison, SPARC was predominantly localised to a separate vesicle population to TGFβig-h3 seen in Fig 5A as distinct green and red punctate staining.

### Dense-core secretory vesicles in MSCs

Based on the findings described above, it was reasoned that it might be possible to identify dense-cored secretory vesicles using TEM. Significantly, therefore, dense-cored secretory vesicles were found in the close vicinity of the Golgi complex (Fig 5B) although similar vesicles were occasionally found scattered throughout the cytosol (diameter 219.9 ± 11.2 nm, n = 37 vesicles from 5 cells in 8 fields; range 173–266 nm). Typically the electron dense core occupied a relatively small proportion of the vesicle. Rough ER and Golgi stacks were observed routinely in all cells.

### Stimulated protein secretion is functionally important

**Fig 6:**
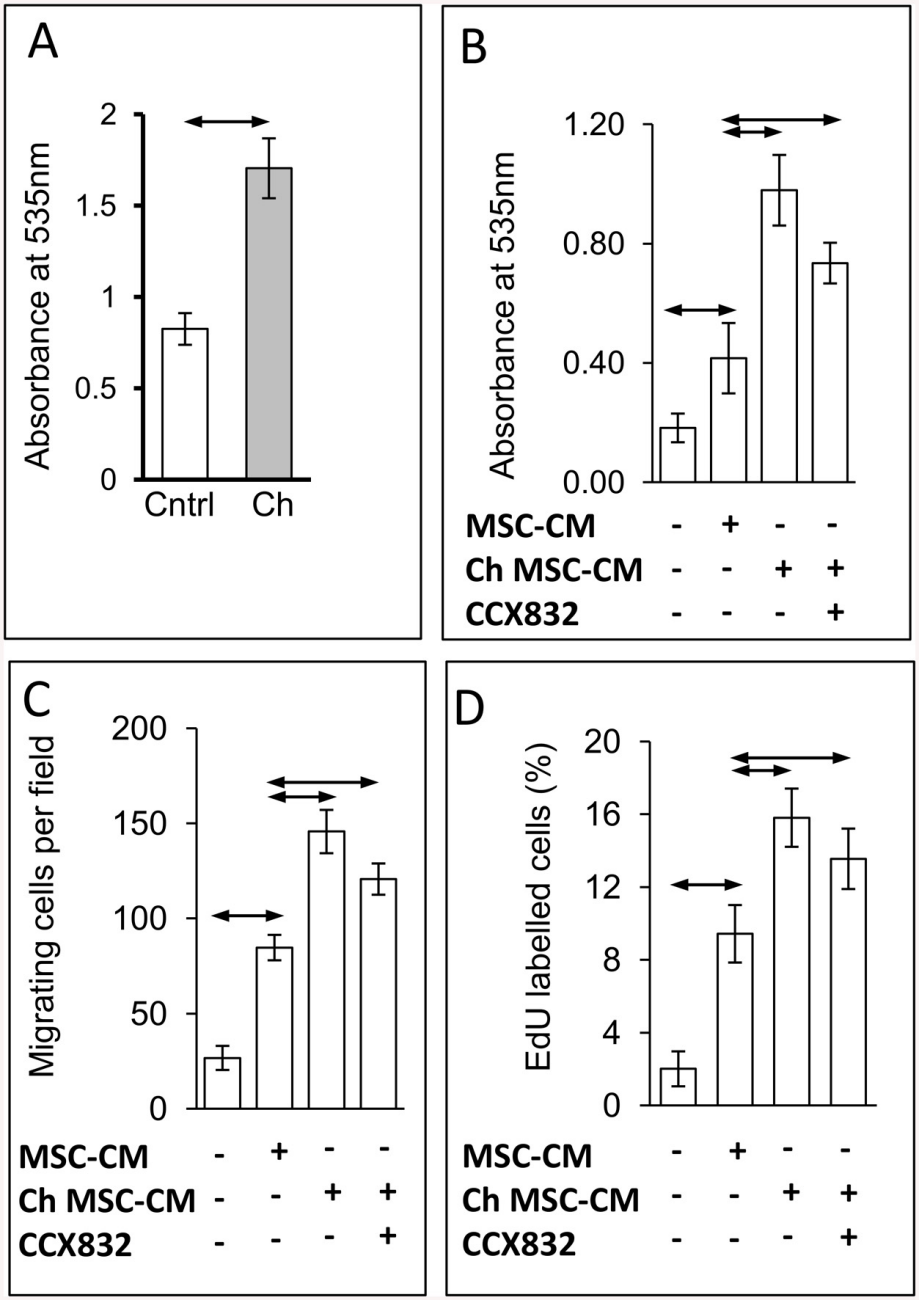
Chemerin-conditioned medium increases cell adhesion, proliferation and migration. *A*. Increased adhesion of MSCs (1x10^5^) after treatment with chemerin (Ch) for 30 min. *B*. Conditioned medium (CM) from MSCs treated with chemerin increased adhesion of normal gastric myofibroblasts and addition of the ChemR23 antagonist CCX832 only slightly reduced the response. *C*. CM from MSCs treated with chemerin stimulated migration of myofibroblasts in Boyden chambers and addition of the ChemR23 antagonist CCX832 only slightly reduced the response. *D*. CM from MSCs treated with chemerin stimulated proliferation of myofibroblasts and addition of the ChemR23 antagonist CCX832 only slightly reduced the response. Means ± SE, n = 3; horizontal arrows, p<0.05.

In order to define the functional significance of regulated exocytosis by MSCs we made use of the data from PANTHER to identify potential functions. Thus, initially we examined the effects of chemerin on MSC adhesion. The latter increased significantly after 30 min chemerin treatment (Fig 6A). We then asked what effect CM from MSCs stimulated with chemerin for 30 min had on adhesion of another stromal cell, the myofibroblast. MSC-CM increased myofibroblast adhesion and this was enhanced still further by pretreatment of MSCs with chemerin for 30min (Fig 6B). To exclude possible actions on myofibroblasts of chemerin transferred in the MSC-CM we treated myofibroblasts with CCX832. The latter slightly, but not significantly reduced the response which remained significantly greater than that to CM from untreated MSCs (Fig 6B). We then used a similar protocol to study the effect of MSC-CM on myofibroblast migration and proliferation. Untreated MSC-CM increased myofibroblast migration and proliferation and chemerin-treated MSC-CM enhanced the response; again, CCX832 treatment of myofibroblasts slightly reduced the responses but these remained significantly greater than those to untreated MSC-CM (Fig 6C and 6D).

## Discussion

The main finding of this study is that two different classes of stimulant, one acting via a GPCR (chemerin) the other via a receptor tyrosine kinase (IGF), are able to trigger exocytosis of a wide range of secretory proteins by MSCs. The mechanism of exocytosis involves a rapid increase in intracellular calcium by influx of extracellular calcium. The stimulated secretion occurs from storage vesicles since neither inhibition of protein synthesis nor of trafficking from the ER reduced the secretory response. A proteomic study of the regulated secretome suggested functional consequences for cell adhesion and we provide evidence that chemerin-stimulated MSC secretion leads to increased adhesion, and also increased adhesion, migration and proliferation of a stromal cell type, the myofibroblast. The data suggest that following recruitment to a tissue, MSCs may rapidly contribute to a change in the cellular microenvironment.

The principle criteria for regulated secretion are (a) the accumulation of secretory product in an intracellular vesicle, (b) secretion in response to stimulation, (c) the secretory response is rapid [16]. The data presented here indicate that protein secretion from MSCs meets all three criteria. Although typically neuronal, endocrine and exocrine cells are associated with regulated secretion, it is nevertheless clear that the same phenotype is also exhibited by other cells including CHO cells and myofibroblasts [16,18,28,29]. In addition, there may be other mechanisms of regulated secretion involving vesicles distinct from those generated at the *trans-*Golgi network and contributing to regulated or constitutive exocytosis [30].

The presence of Ca^2+^ oscillations in a subset of MSCs is well recognised [31], although the basis for the difference between sub-populations of cells remains uncertain. It is also well recognised that microenvironmental signals notably substrate elasticity influence MSC differentiation [32] and that mechanical deformation increases calcium oscillations due to increased calcium influx [33,34]. The present data suggest that Ca^2+^ oscillations are also generated by both growth factors and GPCR agonists, that these depend on extracellular Ca^2+^ and that a consequence of increased intracellular calcium is stimulation of exocytosis. The findings imply that calcium oscillations generated by mechanical stretch might also trigger exocytosis, notably of proteins that influence cell adhesion. The relationship between the microenvironment and MSC function is therefore likely to be a dynamic one with both MSC responses to, and MSC influences on, the microenvironment.

The segregation of proteins destined for secretion by the regulated and constitutive pathways occurs in the *trans-*Golgi network. Two models are frequently employed to account for these processes: “sorting by entry” in which Ca^2+^ and falling pH are thought to cause aggregation of those proteins destined for regulated secretory pathway thereby facilitating their sequestration into vesicle of the regulated pathway [35], and “sorting by retention” in which secretory proteins not destined for secretion are progressively removed from immature regulated secretory vesicles and re-routed to the constitutive pathway [15]. The relevant mechanisms are not necessarily mutually exclusive. Further work will be required to determine the relevant mechanisms in MSCs. It is however, worth noting that in neuroendocrine cells, proteins of the granin family play an important role in this process. A high proportion (96%) of the secretory proteins we identified exhibited rapid release in response to stimulation but we did not find examples of members of the granin family, or for that matter other secretory proteins associated with a neuroendocrine phenotype. In this sense, MSCs differ from another mesenchymal cell type, the myofibroblast, that exhibits regulated exocytosis [18]. There are also differences in the patterns of secretion of proteins that are released by the two cell types. Thus, SPARC did not exhibit regulated exocytosis by MSCs in the present secretome studies, while it had previously been shown to exhibit regulated secretion by gastric myofibroblasts [18]. One protein, IGFBP-7, unexpectedly showed regulated exocytosis in response to IGF-II but not chemerin, and further work will be required to evaluate this finding. Nevertheless, it is evident that the pattern of proteins released by regulated exocytosis varies between mesenchymal cell types and therefore needs to be determined on a cell-by-cell basis.

There is a role for chemerin in differentiation of MSCs, for example in adipogenesis and oesteoclastogenesis [36,37]. The present data suggest that chemerin is also able to acutely influence cell function over periods as short as a few minutes and that these effects can change the cellular environment. There is increasing interest in the role of MSCs both as delivery vehicles in cancer therapy, and in driving cancer progression. The discovery of regulated exocytosis in these cells has implications in both of these instances. Through selective targeting to the secretory pathway it may be possible to ensure delivery of specific proteins in a given cellular environment (for example in response to specific secretagogues) thereby targeting therapy more effectively. Moreover, by understanding the way that MSCs acutely change their behaviour and that of nearby cells, it may be possible to actively promote (in regeneration), or inhibit (in cancer) the interactions of MSC in defined tissue microenvironments.

## Supporting Information

S1 Table (PDF)

S1 Fig (PDF)

S2 Fig (PDF)

S3 Fig (PDF)

S4 Fig (PDF)

S5 Fig (PDF)

S6 Fig (PDF)

## Abbreviations

ECM: extracellular matrix
IGF: insulin-like growth factor
MSC: mesenchymal stromal (stem) cells
