# Supplementary material for: Mesenchymal Stem Cells Exhibit Regulated Exocytosis in Response to Chemerin and IGF
Source: PLoS One. 2015 Oct 29;10(10):e0141331. doi: 10.1371/journal.pone.0141331 (PMC4626093; doi:10.1371/journal.pone.0141331)
Supplement: S1 Table — (PDF) [file pone.0141331.s001.pdf]

Protein Descriptions	First Uniprot ID	Localisation	PEP	Mean ratio IGFII:control	Replicates identified in, of 3
CD59 glycoprotein	P13987	Membrane	8.69E-07	4.0	2
Protein S100-A13	Q99584	Secreted	9.36E-12	3.1	2
Isoform V0 of Versican core protein;Isoform Vint of Versican core protein;Isoform V1 of Vers	P13611	Secreted	3.23E-93	2.7	2
Procollagen C-endopeptidase enhancer 1;cDNA FLJ55126, highly similar to Procollagen C-enc	Q15113	Secreted	1.27E-81	2.6	3
Insulin-like growth factor-binding protein 7	Q16270	Secreted	1.83E-91	2.6	3
Macrophage migration inhibitory factor	P14174	Secreted	7.46E-09	2.6	3
COL6A3 protein;alpha 3 type VI collagen isoform 4 precursor;132 kDa protein	B7ZW00	Secreted	0	2.5	2
Isoform 1 of Tenascin;Isoform 4 of Tenascin;Isoform 3 of Tenascin;Isoform 2 of Tenascin;Isof	P24821	Secreted	1.50E-68	2.5	3
Cathepsin B;cDNA FLJ58073, moderately similar to Cathepsin B;cDNA FLJ40065 fis, clone TES	P07858	Secreted	4.63E-11	2.5	3
Integrin beta-like protein 1;Osteoblast specific cysteine-rich protein;cDNA FLJ59733, highly s	O95965	Secreted	1.10E-23	2.5	3
Isoform 1 of 45 kDa calcium-binding protein;stromal cell derived factor 4 isoform 1 precurso	Q9BRK5	Secreted	1.83E-16	2.4	3
Lumican;23 kDa protein;26 kDa protein	P51884	Secreted	2.48E-90	2.4	3
Galectin-3-binding protein;cDNA FLJ54583, highly similar to Galectin-3-binding protein;cDNA	Q08380	Secreted	3.48E-28	2.3	3
Metalloproteinase inhibitor 2;22 kDa protein;cDNA FLJ57920, highly similar to Metalloprotei	P16035	Secreted	1.14E-34	2.3	3
Bone-derived growth factor (Fragment);Isoform 1 of Sulfhydryl oxidase 1;Putative uncharact	Q13876	Secreted	1.26E-51	2.3	3
Follistatin-related protein 1;cDNA FLJ50214, highly similar to Follistatin-related protein 1	Q12841	Secreted	5.68E-84	2.2	3
Collagen alpha-1(I) chain	P02452	Secreted	0	2.2	3
insulin-like growth factor binding protein 3 isoform a precursor;Insulin-like growth factor-bir	P17936	Secreted	1.22E-09	2.1	3
Collagen alpha-2(I) chain	P08123	Secreted	0	2.1	3
HCG2042771;Tubulin beta-3 chain;HCG1983504, isoform CRA_f;35 kDa protein;46 kDa prote	Q01726	Membrane	1.54E-91	2.1	2
Semaphorin-7A;semaphorin 7A isoform 2 preproprotein;semaphorin 7A isoform 3	O75326	Secreted	3.30E-74	2.1	3
cDNA FLJ54471, highly similar to Complement C1r subcomponent;cDNA FLJ54318, highly sin	P00736	Secreted	1.88E-07	2.1	2
72 kDa type IV collagenase;matrix metalloproteinase 2 isoform b	P08253	Secreted	4.54E-230	2.1	3
Fibrillin-1	P35555	Secreted	0	2.1	3
Annexin A1;cDNA FLJ51887, highly similar to Annexin A1;Annexin A1	P04083	Secreted	5.79E-178	2.1	3
Plasminogen activator inhibitor 1;plasminogen activator inhibitor-1 isoform 2 precursor	P05121	Secreted	1.74E-57	2.0	3
Metalloproteinase inhibitor 1;TIMP metallopeptidase inhibitor 1;TIMP metallopeptidase inhi	P01033	Secreted	4.25E-35	2.0	3
Basement membrane-specific heparan sulfate proteoglycan core protein;Basement membra	P98160	Secreted	0	2.0	3
cDNA FLJ31776 fis, clone NT2RI2008141, highly similar to CALUMENIN;Isoform 1 of Calumen	B3KPG9	Secreted	8.07E-199	2.0	3
plasminogen activator inhibitor type 1, member 2 isoform c precursor;45 kDa protein;Isofor	B4DIF2	Secreted	9.57E-169	2.0	3

Isoform 1 of EGF-containing fibulin-like extracellular matrix protein 1;Isoform 3 of EGF-conta	Q12805	Secreted	7.47E-68	2.0	3
Isoform 2C2 of Collagen alpha-2(VI) chain	P12110	Secreted	7.78E-246	1.9	3
Isoform 1 of Fibronectin;Isoform 3 of Fibronectin;Isoform 5 of Fibronectin;Isoform 15 of Fibr	P02751	Secreted	0	1.9	3
fibronectin 1 isoform 4 preproprotein;FN1 protein;263 kDa protein	P02751-4	Secreted	0	1.9	3
Cathepsin D;Putative uncharacterized protein CTSD;Putative uncharacterized protein CTSD;F	P07339	Secreted	2.02E-45	1.9	3
Isoform 1 of Collagen alpha-3(VI) chain;Isoform 2 of Collagen alpha-3(VI) chain;322 kDa prot	P12111	Secreted	0	1.9	3
Collagen alpha-1(VI) chain	P12109	Secreted	0	1.9	3
Isoform 1 of Collagen alpha-1(XII) chain;Isoform 4 of Collagen alpha-1(XII) chain	Q99715	Secreted	0	1.8	3
clust	P07355	Secreted	3.62E-273	1.8	3
Collagen alpha-1(V) chain	P20908	Secreted	1.17E-31	1.8	3
Galectin-1	P09382	Secreted	2.09E-117	1.8	3
Isoform 1 of Tissue-type plasminogen activator;Isoform 3 of Tissue-type plasminogen activat	P00750	Secreted	2.61E-60	1.8	2
Transforming growth factor-beta-induced protein ig-h3;60 kDa protein;Transforming growth	Q15582	Secreted	1.51E-140	1.7	3
Aminopeptidase N	P15144	Membrane	1.09E-27	1.7	3
Periostin, osteoblast specific factor;Isoform 4 of Periostin	B1ALD9	Secreted	4.25E-127	1.7	3
Annexin A5;Putative uncharacterized protein ANXA5 (Fragment)	P08758	Secreted	2.87E-165	1.7	3
Isoform A of Decorin;Isoform D of Decorin	P07585	Secreted	4.36E-17	1.7	3
Protein disulfide-isomerase;cDNA FLJ59430, highly similar to Protein disulfide-isomerase;Put	P07237	Membrane	7.29E-104	1.7	3
Protein-lysine 6-oxidase	P28300	Secreted	3.66E-59	1.7	3
Target of Nesh-SH3 precursor (Tarsh) (Nesh-binding protein) (NeshBP) (ABI gene family mem	Q7Z7G0	Secreted	1.71E-26	1.7	2
Metalloproteinase inhibitor 3;TIMP metallopeptidase inhibitor 3	P35625	Secreted	4.43E-14	1.7	3
Testican-1	Q08629	Secreted	5.49E-07	1.6	2
Insulin-like growth factor-binding protein 4	P22692	Secreted	3.26E-07	1.6	2
Lactadherin;milk fat globule-EGF factor 8 protein isoform b	Q08431	Membrane	9.23E-11	1.6	3
Collagen alpha-2(V) chain;110 kDa protein;COL5A2 protein;cDNA FLJ53096, highly similar to	P05997	Secreted	4.11E-39	1.5	3
Pentraxin-related protein PTX3	P26022	Secreted	1.28E-82	1.5	3
Biglycan;cDNA FLJ35635 fis, clone SPLEN2011805, highly similar to BONE/CARTILAGE PROTE	P21810	Secreted	1.48E-31	1.5	3
Thrombospondin-1	P07996	Secreted	0	1.5	3
Isoform 2 of Clusterin;Isoform 1 of Clusterin;CLU;54 kDa protein	P10909	Secreted	3.15E-52	1.4	3
Isoform 1 of Connective tissue growth factor;Isoform 2 of Connective tissue growth factor	P29279	Secreted	1.20E-56	1.4	3
Lysyl oxidase homolog 2;cDNA FLJ53707, highly similar to Lysyl oxidase homolog 2	Q9Y4K0	Secreted	1.77E-91	1.3	3
Serine protease 23;cDNA FLJ51190, highly similar to Serine protease 23	O95084	Secreted	3.83E-35	1.2	2
SPARC	P09486	Secreted	3.09E-71	1.2	3

Latent-transforming growth factor beta-binding protein 2	Q14767	Secreted	5.98E-139	0.8	2

Table S1. Secretory proteins quantified in the media of IGF-treated and control MSCs. The Table identifies the names of secreted proteins (ie typically those with a signal sequence) identified in the media of MSCs after SILAC labelling followed by treatment with or without IGF-II for 30min. The first Uniprot ID, the PEP score, relative abundance in IGF vs control media, and number of replicates of three in which two or more tryptic peptides were found. IGF-II itself meets the criteria for identification but is omitted from the Table. The list is ranked by the fold-change in response to IGF-II. Proteins validated by western blot are highlighted.
