# Supplementary material for: Mesenchymal Stem Cells Exhibit Regulated Exocytosis in Response to Chemerin and IGF
Source: PLoS One. 2015 Oct 29;10(10):e0141331. doi: 10.1371/journal.pone.0141331 (PMC4626093; doi:10.1371/journal.pone.0141331)
Supplement: S1 Fig — (PDF) [file pone.0141331.s002.pdf]

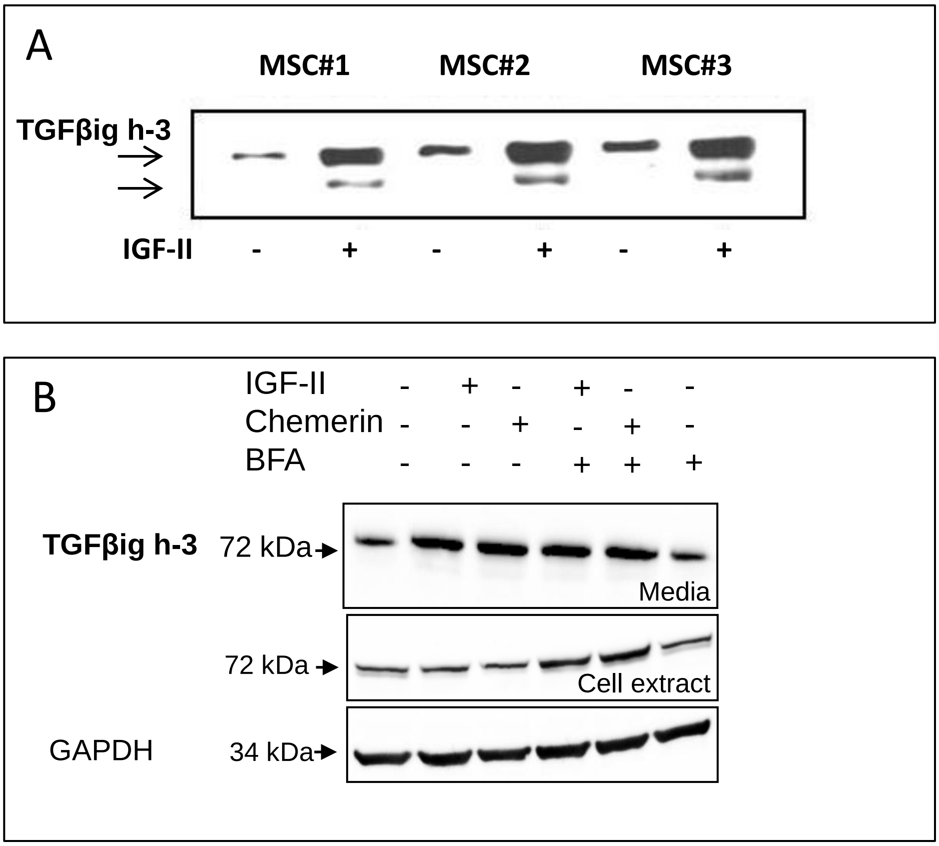

S1 Fig. Regulated secretion of TGFβig-h3 by MSCs. *A.* Representative **w**estern blots of TGFβig-h3 in media from three MSC lines all exhibiting increased TGFβig-h3 secretion after IGF-II (100ng.ml^{-1}) (mean fold increase 3.6 ± 0.6 n = 6). *B.* Chemerin- and IGF-II-stimulated release of TGFβig-h3 was not effected by brefeldin A (BFA) treatment. There was a small increase in TGFβig-h3 in cell extracts of BFA treated cells stimulated with chemerin and IGF-II but no change in GAPDH.
