# Supplementary material for: Mesenchymal Stem Cells Exhibit Regulated Exocytosis in Response to Chemerin and IGF
Source: PLoS One. 2015 Oct 29;10(10):e0141331. doi: 10.1371/journal.pone.0141331 (PMC4626093; doi:10.1371/journal.pone.0141331)
Supplement: S2 Fig — (PDF) [file pone.0141331.s003.pdf]

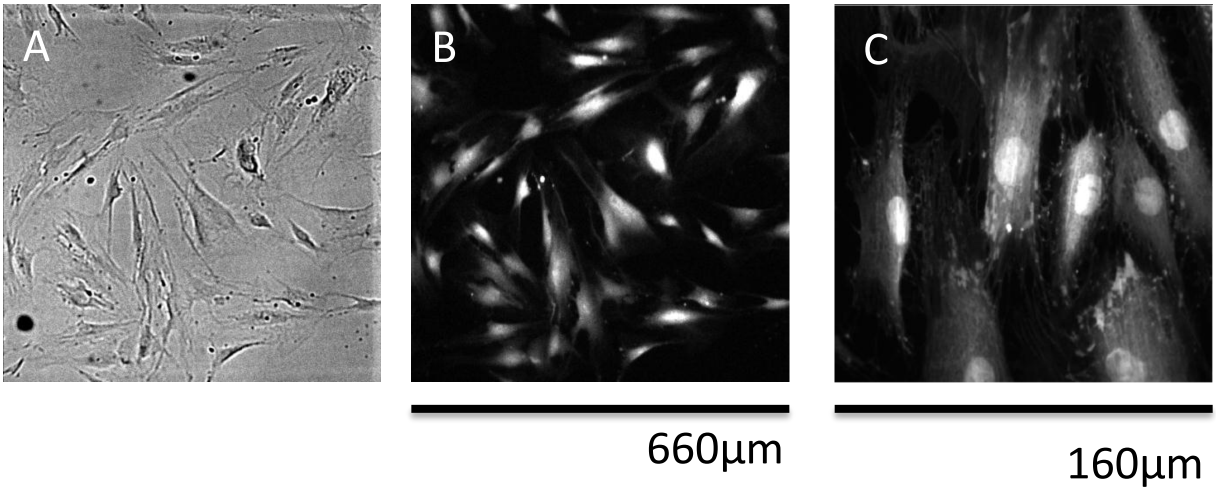

S2 Fig. Images of Fluo-4 labelled MSCs. *A.* Transmitted, *B*, grey scale image of Fluo-4 loaded control MSCs, and C, grey scale image of a chemerin-treated MSC, taken at x10 magnification (A, B) and (C) x40 magnification.
