# Supplementary material for: Mesenchymal Stem Cells Exhibit Regulated Exocytosis in Response to Chemerin and IGF
Source: PLoS One. 2015 Oct 29;10(10):e0141331. doi: 10.1371/journal.pone.0141331 (PMC4626093; doi:10.1371/journal.pone.0141331)
Supplement: S3 Fig — (PDF) [file pone.0141331.s004.pdf]

(A) IGFBP7

(i)

```
        10         20         30         40         50         60         70         80
MERPSLRALL LGAAGLLLLL LPLSSSSSSD TCGPCEPASC PPLPPLGCLL GETRDACGCC PMCARGEGEP CGGGGAGRGY

        90        100        110        120        130        140        150        160
CAPGMECVKS RKRRKGKAGA AAGGPGVSGV CVCKSRYPVC GSDGTTYPSG CQLRAASQRA ESRGEKAITQ VSKGTCEQGP

       170        180        190        200        210        220        230        240
SIVTPPKDIW NVTGAQVYLS CEVIGIPTPV LIWNKVKRGH YGVQRTELLP GDRDNLAIQT RGGPEKHEVT GWVLVSPLSK

       250        260        270        280
EDAGEYECHA SNSQGQASAS AKITVVDALH EIPVKKGEGA EL
```

(ii)

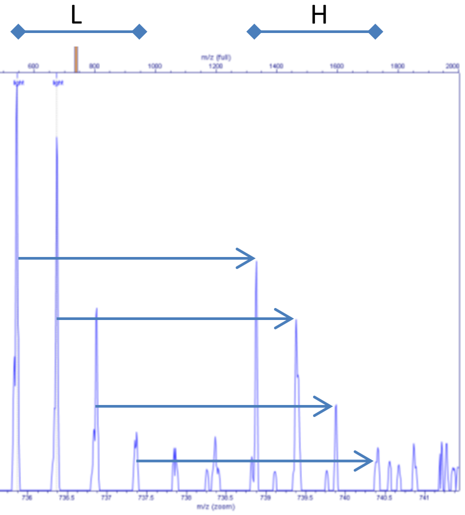

Peptide: GTCEQGPSIVTPPK

(iii)

Sequence	Mean ratio IGF-II:Control	Peptide ion score
AGAAAGGPGVSGVCVCK	2.2	237.6
DNLAIQTR	2.9	131.47
EDAGEYECHASNSQGQASASAK	1.9	196.56
GEGEPCGGGGAGR	2.4	159.88
GHYGVQR	2.3	84.728
GTCEQGPSIVTPPK	2.4	195.75
GYCAPGMECVK	3.0	130
HEVTGWVLVSPLSK	0.9	162.05
ITVVDALHEIPVK	2.9	226.1
TELLPGDR	3.0	91.683
TELLPGDRDNLAIQTR	3.3	97.716

(B) Lumican

(i)

```
        10         20         30         40         50         60         70         80
MSLSAFTLFL ALIGGTSGQY YDYDFPLSIY GQSSPNCAPE CNCPESYPSA MYCDELKLKS VPMVPPGIKY LYLRNNQIDH

        90        100        110        120        130        140        150        160
IDEKAFENVT DLQWLILDHN LLENSKIKGR VFSKLKQLKK LHINHNNLTE SVGPLPKSLE DLQLTHNKIT KLGSFEGLVN

       170        180        190        200        210        220        230        240
LTFIHLQHNR LKEDAVSAAF KGLKSLEYLD LSFNQIARLP SGLPVSLLTL YLDNNKISNI PDEYFKRFNA LQYLRLSHNE

       250        260        270        280        290        300        310        320
LADSGIPGNS FNVSSLVELD LSYNKLKNIP TVNENLENYY LEVNQLEKFD IKSFCKILGP LSYSKIKHLR LDGNRISETS

       330
LPPDMYECLR VANEVTLN
```

(ii)

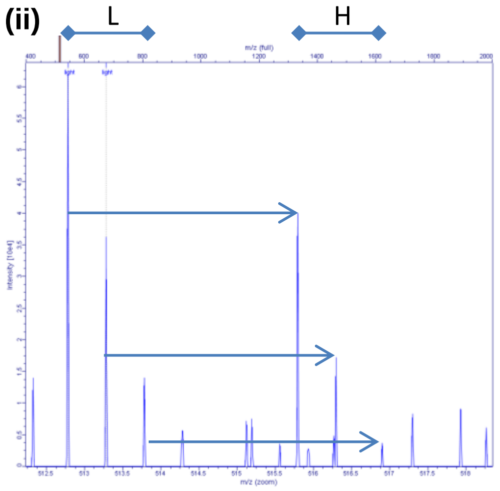

Peptide: FNALQYLR

(iii)

Sequence	Mean ratio IGF-II:Control	Peptide ion score
FNALQYLR	2.84	164.4
ILGPLSYSK	2.70	81.136
LKEDAVSAAFK	3.76	140.91
NNQIDHIDEK	2.20	184.6
SLEDLQLTHNK	2.08	276.93
SLEYLDLSFNQIAR	1.73	294.15

(C) SPARC

(i)

```
        10         20         30         40         50         60         70         80
MRAWIFFLLC LAGRALAAPQ QEALPDETEV VEETVAEVTE VSVGANPVQV EVGEFDDGAE ETEEEVVAEN PCQNHHCKHG

        90        100        110        120        130        140        150        160
KVCELDENNT PMCVCQDPTS CPAPIGEFEK VCSNDNKTFD SSCHFFATKC TLEGTKKGHK LHLDYIGPCK YIPPCLDSEL

       170        180        190        200        210        220        230        240
TEFPLRMRDW LKNVLVTLYE RDEDNNLLTE KQKLRVKKIH ENEKRLEAGD HPVELLARDF EKNYNMYIFP VHWQFGQLDQ

       250        260        270        280        290        300
HPIDGYLSHT ELAPLRAPLI PMEHCTTRFF ETCDLDNDKY IALDEWAGCF GIKQKDIDKD LVI
```

(ii)

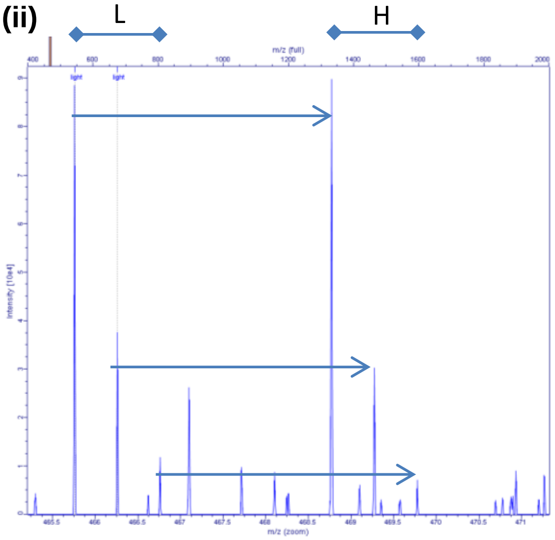

Peptide: DIDKDLVI

(iii)

Sequence	Mean ratio IGF-II:Control	Peptide ion score
DEDNNLLTEK	1.3	188.92
DIDKDLVI	1.3	108.85
FFETCDLDNDK	1.1	196.89
LEAGDHPVELLAR	1.1	225.35
LHLDYIGPCK	1.2	139.33
TFDSSCHFFATK	1.2	220.65
YIALDEWAGCFGIK	1.2	222.79
YIPPCLDSELTEFPLR	1.2	116.97

(D) PAI-1

(i)

```
         10         20         30         40         50         60         70         80
MQMSPALTCL VLGLALVFGE GSAVHHPPSY VAHLASDFGV RVFQQVAQAS KDRNVVFSPY GVASVLAMLQ LTTGGETQQQ

         90        100        110        120        130        140        150        160
IQAAMGFKID DKGMAPALRH LYKELMGPWN KDEISTTDAI FVQRDLKLVQ GFMPHFFRLF RSTVKQVDFS EVERARFIIN

        170        180        190        200        210        220        230        240
DWVKTHTKGM ISNLLGKGAV DQLTRLVLVN ALYFNGQWKT PFPDSSTHRR LFHKSDGSTV SVPMMAQTNK FNYTEFTTPD

        250        260        270        280        290        300        310        320
GHYYDILELP YHGDTLSMFI AAPYEKEVPL SALTNILSAQ LISHWKGNMT RLPRLLVLPK FSLETEVDLR KPLENLGMTD

        330        340        350        360        370        380        390        400
MFRQFQADFT SLSDQEPLHV AQALQKVKIE VNESGTVASS STAVIVSARM APEEIIMDRP FLFVVRHNPT GTVLFMGQVM EP
```

(ii)

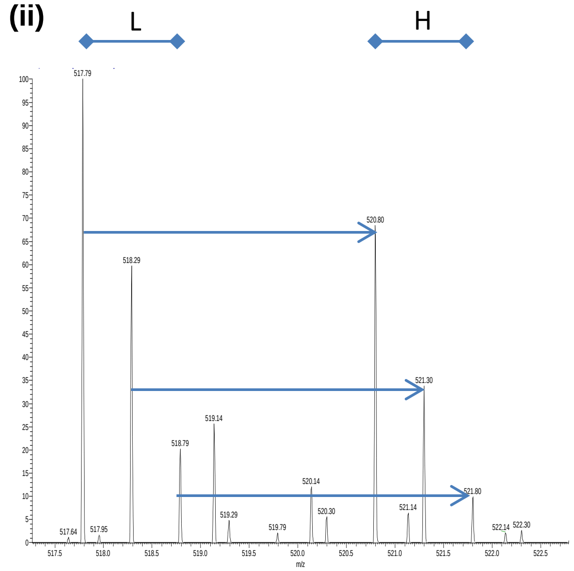

Peptide: FIINDWVK

(iii)

Sequence	Mean ratio IGF-II:Control	Peptide ion score
VFQQVAQASK	2.3	197.55
FIINDWVK	2.2	183.9
FSLETEVDLR	1.6	179.33
GAVDQLTR	3.0	125.39
GMISNLLGK	2.3	125.44
HNPTGTVLFMGQVMEP	5.8	142.58
PFLFVVR	2.0	104.82
QVDFSEVER	1.8	123.43
SDGSTVSVPMMAQTNK	2.0	188.33
TPFPDSSTHR	0.7	92.192

(E) TIMP1

(i)

```
        10         20         30         40         50         60         70         80
MAPFEPLASG ILLLLWLIAP SRACTCVPPH PQTAFCNSDL VIRAKFVGTP EVNQTTLYQR YEIKMTKMYK GFQALGDAAD

        90        100        110        120        130        140        150        160
IRFVYTPAME SVCGYFHRSH NRSEEFLIAG KLQDGLLHIT TCSFVAPWNS LSLAQRRGFT KTYTVGCEEC TVFPCLSIPC

       170        180        190        200
KLQSGTHCLW TDQLLQGSEK GFQSRHLACL PREPGLCTWQ SLRSQIA
```

(ii)

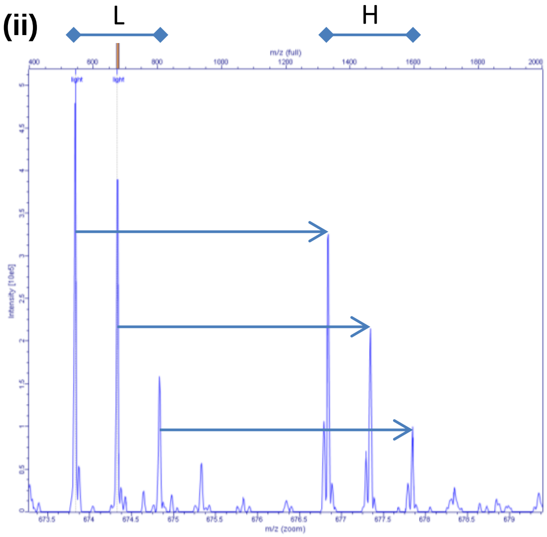

Peptide: EPGLCTWQSLR

(iii)

Sequence	Mean ratio IGF-II:Control	Peptide ion score
EPGLCTWQSLR	1.7	87.125
FVYTPAMESVCGYFHR	1.9	88.09
GFQALGDAADIR	1.8	229
HLACLPR	2.1	76.403
SEEFLIAGK	2.1	191.53

(F) MMP-2

(i)

```
 10         20         30         40         50         60         70         80         90
MEALMARGAL TGPLRALCLL GCLLSHAAAA PSPIIKFPGD VAPKTDKELA VQYLNTFYGC PKESCNLFVL KDTLKKMQKF FGLPQTGDLD

       100        110        120        130        140        150        160        170        180
QNTIETMRKP RCGNPDVANY NFFPRKPKWD KNQITYRIIG YTPDLDPETV DDAFARAFQV WSDVTPLRFS RIHDGEADIM INFGRWEHGD

       190        200        210        220        230        240        250        260        270
GYPFDGKDGL LAHAFAPGTG VGGDSHFDDD ELWTLGEGQV VRVKYGNADG EYCKFPFLFN GKEYNSCTDT GRSDGFLWCS TTYNFEKDGK

       280        290        300        310        320        330        340        350        360
YGFCPHEALF TMGGNAEGQP CKFPFRFQGT SYDSCTTEGR TDGYRWCGTT EDYDRDKKYG FCPETAMSTV GGNSEGAPCV FPFTFLGNKY

       370        380        390        400        410        420        430        440        450
ESCTSAGRSD GKMWCATTAN YDDDRKWGFC PDQGYSLFLV AAHEFGHAMG LEHSQDPGAL MAPIYTYTKN FRLSQDDIKG IQELYGASPD

       460        470        480        490        500        510        520        530        540
IDLGTGPTPT LGPVTPEICK QDIVFDGIAQ IRGEIFFFKD RFIWRTVTPR DKPMGPLLVA TFWPELPEKI DAVYEAPQEE KAVFFAGNEY

       550        560        570        580        590        600        610        620        630
WIYSASTLER GYPKPLTSLG LPPDVQRVDA AFNWSKNKKT YIFAGDKFWR YNEVKKKMDP GFPKLIADAW NAIPDNLDAV VDLQGGGHSY

       640        650        660
FFKGAYYLKL ENQSLKSVKF GSIKSDWLGC
```

(ii)

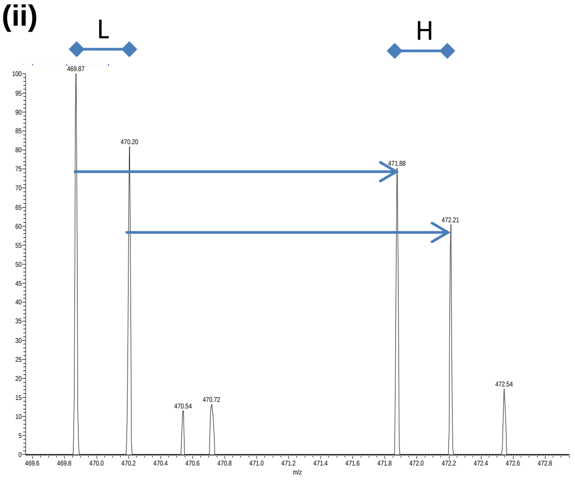

Peptide: WEHGDGYPFDGK

(iii)

Sequence	Mean ratio IGFII:control	Peptide Score
AFQVWSDVTPLR	2.0	208.56
ESCNLFVLK	2.2	137.97
EYNSCTDTGR	1.3	142.86
FPFLFNGK	2.4	120.59
FQGTSYDSCTTEGR	2.1	265.08
IDAVYEAPQEEK	2.1	193.52
IIGYTPDLDPETVDDAFAR	2.4	249.51
LENQSLK	1.8	115.33
MWCATTANYDDDR	1.7	271.83
QDIVFDGIAQIR	1.7	249.91
SDGFLWCSTTYNFEK	1.9	240.82
TYIFAGDK	1.9	108
VDAAFNWSK	2.2	174.76
WCGTTEDYDR	2.0	151.16
WEHGDGYPFDGK	2.5	123.46
YESCTSAGR	1.8	155.32
YGNADGEYCK	2.1	121.87

(G) TGFβig-h3

(i)

```
        10         20         30         40         50         60         70         80         90
MALFVRLLAL ALALALGPAA TLAGPAKSPY QLVLQHSRLR GRQHGPNVCA VQKVIGTNRK YFTNCKQWYQ RKICGKSTVI SYECCPGYEK

       100        110        120        130        140        150        160        170        180
VPGEKGCPAA LPLSNLYETL GVVGSTTTQL YTDRTEKLRP EMEGPGSFTI FAPSNEAWAS LPAEVLDSLV SNVNIELLNA LRYHMVGRRV

       190        200        210        220        230        240        250        260        270
LTDELKHGMT LTSMYQNSNI QIHHYPNGIV TVNCARLLKA DHHATNGVVH LIDKVISTIT NNIQQIIEIE DTFETLRAAV AASGLNTMLE

       280        290        300        310        320        330        340        350        360
GNGQYTLLAP TNEAFEKIPS ETLNRILGDP EALRDLLNNH ILKSAMCAEA IVAGLSVETL EGTTLEVGCS GDMLTINGKA IISNKDILAT

       370        380        390        400        410        420        430        440        450
NGVIHYIDEL LIPDSAKTLF ELAAESDVST AIDLFRQAGL GNHLSGSERL TLLAPLNSVF KDGTPPIDAH TRNLLRNHII KDQLASKYLY

       460        470        480        490        500        510        520        530        540
HGQTLETLGG KKLRVFVYRN SLCIENSCIA AHDKRGRYGT LFTMDRVLTP PMGTVMDVLK GDNRFSMLVA AIQSAGLTET LNREGVYTVF

       550        560        570        580        590        600        610        620        630
APTNEAFRAL PPRERSRLLG DAKELANILK YHIGDEILVS GGIGALVRLK SLQGDKLEVS LKNNVVSVNK EPVAEPDIMA TNGVVHVITN

       640        650        660        670        680
VLQPPANRPQ ERGDELADSA LEIFKQASAF SRASQRSVRL APVYQKLLER MKH
```

(ii)

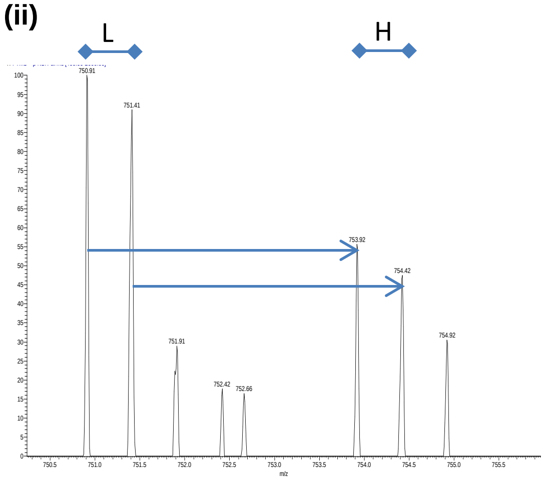

Peptide: VLTPPMGTVMDVLK

(iii)

Sequence	Mean ratio IGFII:control	Peptide Score
STVISYECCPGYEK	1.7	262.55
STVISYECCPGYEKVPGEK	1.8	117.87
VLTDELK	2.1	111.67
YFTNCK	0.9	62.75
DLLNNHILK	1.8	145.23
EGVYTVFAPTNEAFR	1.9	216.6
GDELADSALEIFK	1.7	233.31
ILGDPEALR	1.8	131.05
IPSETLNR	1.8	135.65
LTLLAPLNSVFK	1.7	177.01
NSLCIENSCIAAHDK	1.7	266.78
NSLCIENSCIAAHDKR	1.6	91.886
QAGLGNHLSGSER	1.8	121.08
SLQGDKLEVSLK	1.7	193.89
VLTPPMGTVMDVLK	1.7	161.27
YGTLFTMDR	1.2	161.75
YLYHGQTLETLGGK	1.7	216.05

(H) MIF

(i)

```
         10         20         30         40         50         60         70         80
MPMFIVNTNV PRASVPDGFL SELTQQLAQA TGKPPQYIAV HVVPDQLMAF GGSSEPCALC SLHSIGKIGG AQNRSYSKLL

90         100        110
CGLLAERLRI SPDRVYINYY DMNAANVGWN NSTFA
```

(ii)

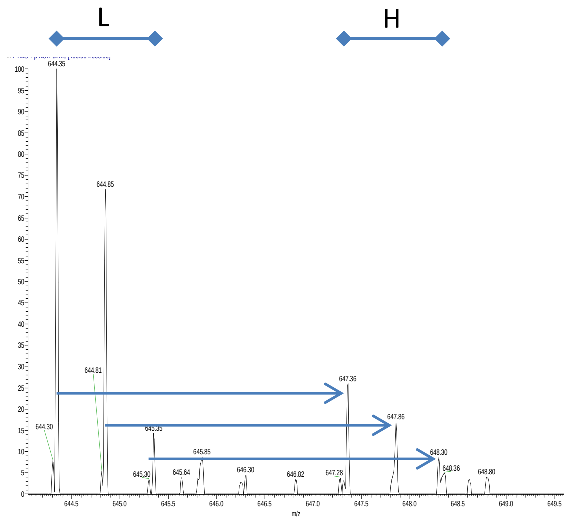

Peptide: PMFIVNTNVPR

(iii)

Sequence	Mean ratio IGFII:control	Peptide Score
LLCGLLAER	2.4	161.6
PMFIVNTNVPR	2.4	163.04

S3 Fig. Examples of SILAC identification of MSC secretome proteins. *A*. IGFBP-7; *B*, lumican; *C*, SPARC; *D*, PAI-1; *E*, TIMP-1, *F*, MMP-2, *G*, TGFβig-h3, *H*, MIF. The data for each protein are presented in the same format. (*i*). The protein sequence indicating by highlighting the tryptic peptides identified in at least 2 out of 3 experimental replicates and by a double headed arrow the peptide illustrated in (ii). (ii) Representative spectrum showing peaks corresponding to matching light (L) and heavy (H) ions indicated by arrows. (iii) A table listing the tryptic peptides identified, the ratio of IGF-II to control, and the peptide ion score.
