# Supplementary material for: Mesenchymal Stem Cells Exhibit Regulated Exocytosis in Response to Chemerin and IGF
Source: PLoS One. 2015 Oct 29;10(10):e0141331. doi: 10.1371/journal.pone.0141331 (PMC4626093; doi:10.1371/journal.pone.0141331)
Supplement: S4 Fig — (PDF) [file pone.0141331.s005.pdf]

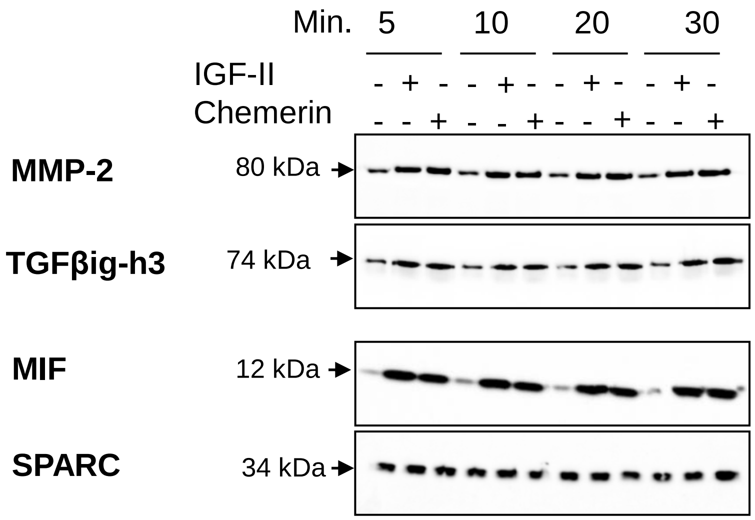

S4 Fig. Chemerin and IGF-II stimulate protein secretion within 5 min. Western blots of media of MSCs stimulated for 5, 10, 20 or 30 min with IGF-II or chemerin. Note stimulated secretion of three representative proteins that exhibit regulated exocytosis (MMP-2, TGFβig-h3, and MIF) but of not of SPARC.
