# Supplementary material for: Mesenchymal Stem Cells Exhibit Regulated Exocytosis in Response to Chemerin and IGF
Source: PLoS One. 2015 Oct 29;10(10):e0141331. doi: 10.1371/journal.pone.0141331 (PMC4626093; doi:10.1371/journal.pone.0141331)
Supplement: S5 Fig — (PDF) [file pone.0141331.s006.pdf]

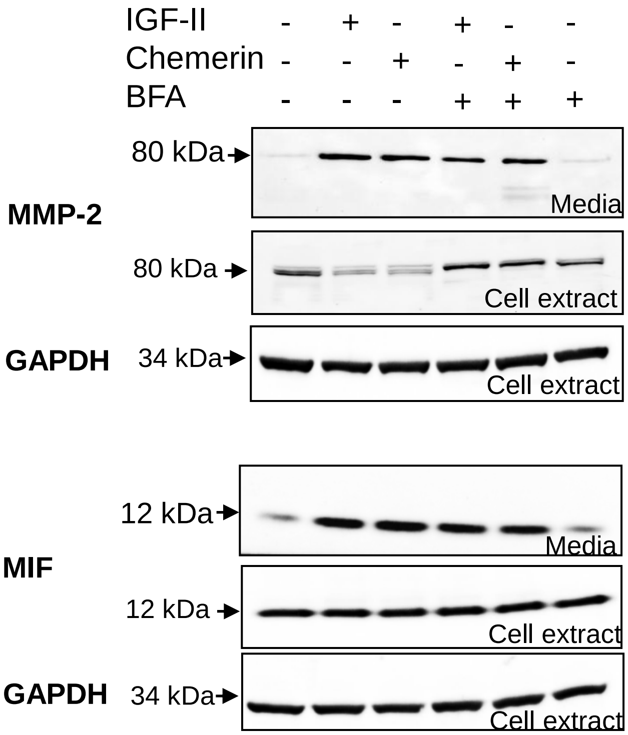

S5 Fig. Chemerin- and IGF-II-stimulated protein secretion is resistant to BFA. Examples of two protein exhibiting stimulated secretion (MMP-2, MIF) showing in both cases that stimulated secretion is resistant to BFA. Note the latter does, however, show an accumulation of MMP-2 in cell extracts compared with GAPDH as a control.
