# Supplementary material for: Mesenchymal Stem Cells Exhibit Regulated Exocytosis in Response to Chemerin and IGF
Source: PLoS One. 2015 Oct 29;10(10):e0141331. doi: 10.1371/journal.pone.0141331 (PMC4626093; doi:10.1371/journal.pone.0141331)
Supplement: S6 Fig — (PDF) [file pone.0141331.s007.pdf]

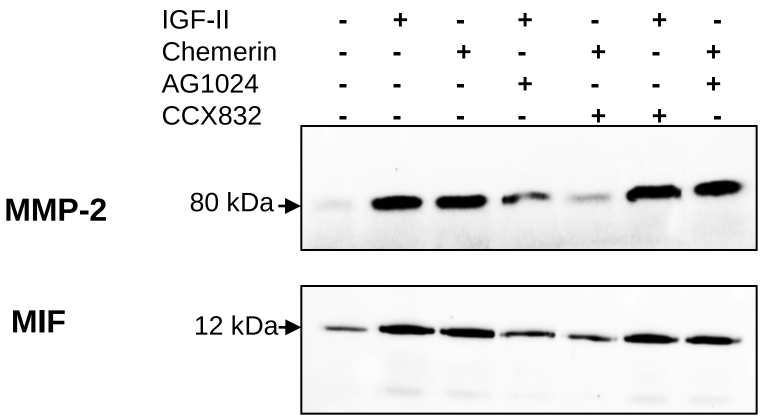

S6 Fig. Inhibitors of chemR23 and IGF-R reduced the stimulated-release of proteins in response to chemerin and IGF-II, respectively, but not vice versa. Representative western blots of media for two proteins exhibiting regulated secretion showing specificity of receptor mechanisms.
